# Dental implant location via surface scanner: a pilot study

**DOI:** 10.1186/s12903-020-01297-y

**Published:** 2020-11-04

**Authors:** Miao Zhou, Hui Zhou, Shu-yi Li, Yuan-ming Geng

**Affiliations:** 1 Department of Oral and Maxillofacial Surgery, Affiliated Stomatology Hospital of Guangzhou Medical University, Guangzhou Key Laboratory of Basic and Applied Research of Oral Regenerative Medicine, Huangshadadao Road 39, Guangzhou, 510182 China; 2 Department of Stomatology, The Eighth People Hospital of Guangzhou, Huayinglu Road 8, Guangzhou, 510060 China; 3 Department of Stomatology, Zhujiang Hospital, Southern Medical University, Gongyedadaozhong Road 253, Guangzhou, 510282 China

**Keywords:** Dental implant, Accuracy, Digital impression, Intraoral scanner, Extraoral scanner, Cone beam computerized tomography

## Abstract

**Purpose:**

Implant location is performed after placement to verify that the safety of neighboring anatomic structure and the realizability of prosthetic plan. Routine postoperative location is based on radiological scanning and raises the concerns on radiation exposure and inconveniency in practice. In the present study a location method based on surface scanning was introduced and the accuracy of this method was assessed in vitro.

**Material and methods:**

A total of 40 implants were placed in 10 resin mandible models. The models were scanned with intraoral scanner (IS group) and extraoral scanner (ES group). The implant position was located with fusing the images of surface scanning and cone beam computerized tomography (CBCT) after implant placement. Deviations were measured between positions located by surface scanner and postoperative CBCT with the parameters: central deviation at apex (cda), central deviation at hex (cdh), horizontal deviation at apex (hda), horizontal deviation at hex (hdh), vertical deviation at apex (vda), vertical deviation at hex (vdh) and angular deviation (ad).

**Results:**

In IS group, the mean value of cda, cdh, hda, hdh, vda, vdh and ad was 0.27 mm, 0.23 mm, 0.12 mm, 0.10 mm, 0.21 mm, 0.19 mm and 0.72°, respectively. In ES group, the mean value of cda, cdh, hda, hdh, vda, vdh and ad was 0.28 mm, 0.25 mm 0.14 mm, 0.11 mm, 0.22 mm, 0.20 mm and 0.68°, respectively. The implant deviations in IS and ES groups were of no significant difference for any of the measurements.

**Conclusions:**

Dental implant can be located via surface scanner with acceptable accuracy for postoperative verification. Further clinical investigation is needed to assess the feasibility of the method.

## Backgrounds

Although the position of oral implant could be planned quite perfectly before surgery, implant deviation is inevitable regardless of guidance strategy and operator experience [1, 2]. Postoperative examination is required routinely to confirm that the critical anatomical structures are well preserved, and the implant is placed in the planned position that favors the following prosthetic restoration.

For present, the methods to assess the inserted implant are mostly based on radiology, such as intraoral radiography, panoramic radiography and cone beam computed tomography (CBCT). CBCT offers the three-dimensional view on the implant and adjacent tissue, and is thought to be the most accurate way to locate the implant position [3]. But due to the consideration on radiation safety, dental radiological devices have to be kept in a separate room with protective barrier. For this reason, the examination is normally performed after the whole surgery is completed and the patient leaves the operation room. If unacceptable deviation occurs, it’s would be a difficult decision for the surgeon whether a corrective reentry is necessary. Another drawback of postoperative radiography is that the patient’s acceptability is often limited due to the growing concern on radiation hazard [4].

Alternative methods to locate implant postoperatively included measuring the difference in the angles of virtual planned and definitively used abutment replicas on the working model [5], or fusing the preoperative planned implant with the postoperative CBCT scans of the implant replicas of model cast [6]. These methods are unable to tell the surgeon the exact position of implant immediately after insertion and don’t allow any adjustment if the implant is not in a favorable position, because they generally require conventional impression and cast production, which are quite time-consuming.

A more simplified solution could be the introduction of digital impression. Digital impression is a three-dimensional model reconstructed from the data collected by a surface scanner, which records the geometry of tissue surface by measuring the light reflection times of the subject surface. The whole procedure usually costs within few minutes [7]. With known and mature digital tools, digital impression can be fused with CBCT images by matching fiducial points, such as remaining tooth, temporary implant and fixation pin [8]. Considering that the spatial relation of fiducial points to local anatomical structure is constant in a certain period, it’s possible to assess the position of placed implant to neighboring tissue by measuring the its relation to fiducial points. The digital impression with placed implants can also be directly used in following design and fabrication of immediate dental prosthetics, if the implant is in the ideal position. In this workflow, the surgical and restorative phases of the treatment would be both greatly simplified. Furthermore, intraoperative adjustment of deviated implant becomes possible if a handheld device is used, most mainstream commercial intraoral scanners for example.

However, in order to include fiducial points that are distributed dispersedly, a full arch scan is necessary to assess the position of placed implant. Although the precision of surface scanning devices has improved considerably over these years, and now is acceptable for crowns and short fixed prostheses [9, 10], the devices are still vulnerable to inaccuracies in scanning of increased span [11]. Whether the accuracy via surface scanner fulfills the needs in implant location is questionable.

In this study, the authors attempted to assess the implant position by matching scan body that was virtual-connected to implant with the surface scanning image. The accuracy of location was assessed by comparison with postoperative CBCT examination.

## Materials and methods

### Implant placement

A mandible model with bilateral second premolar and first molar missing was designed based on the CBCT data of a volunteer’s mandible in the software (Mimics v16.0, Materialise, Leuven, Belgium). A total of ten resin replicas of the designing were fabricated using rapid prototyping (ProJet 3500 HD MAX, 3D System, Rock Hill, SC, USA). Four implants (Straumann Standard Plus RN, 4.1 × 10 mm, Straumann AG, Basel, Switzerland) were placed in the edentulous regions of each model. The models were scanned with CBCT (3D eXam, Kavo, Bismarckring, Germany) postoperatively.

### Implant location

**Fig. 1:**
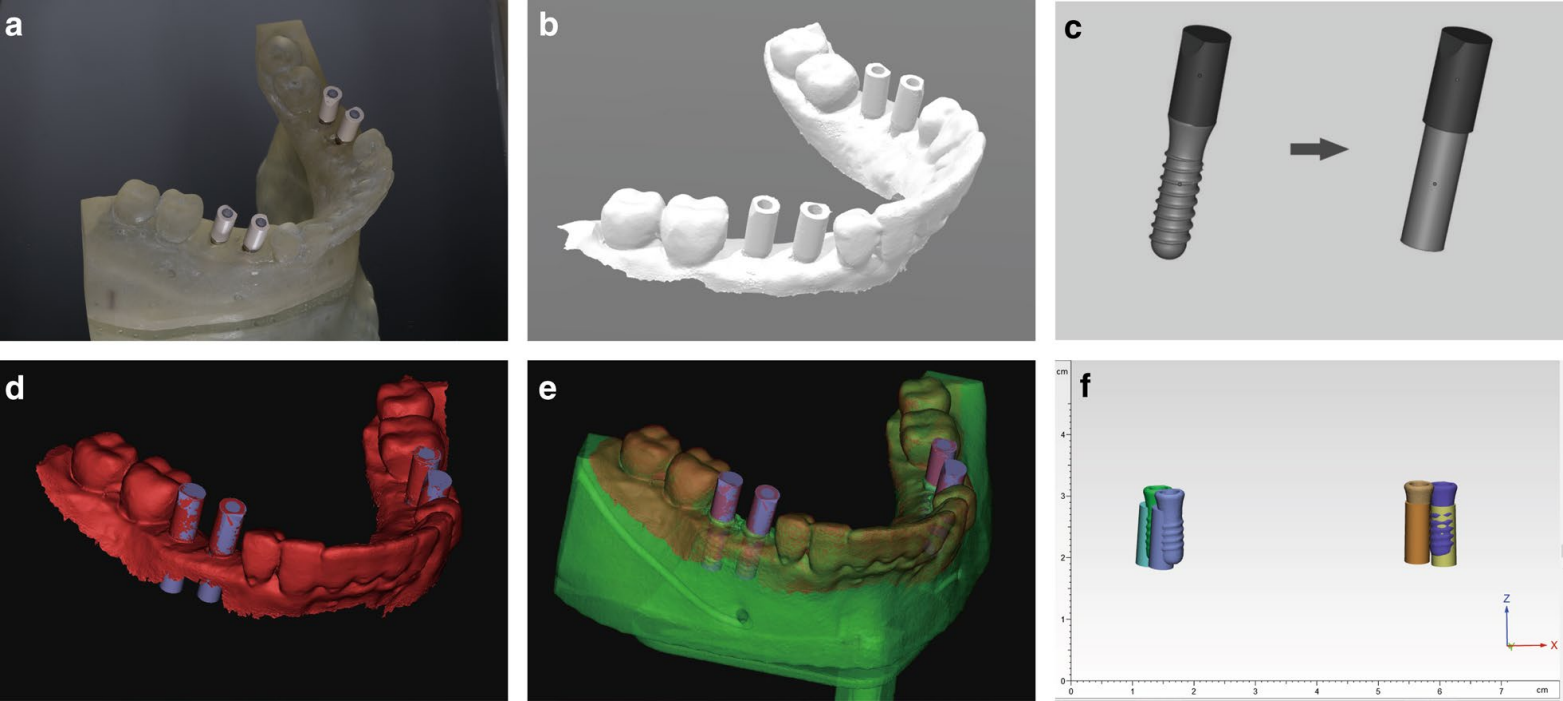
Dental implant location via surface scanner. **a** Scan bodies were connected to the implants placed in the resin mandible model; **b** the model was scanned with intraoral and extraoral scanners; **c** location component: scan body and implant were connected virtually and the implant part was simplified to a cylinder of same diameter and length; **d** The location component was aligned in the surface scanning image by matching the geometry of scan body; **e** the resulted model in (**d**) was aligned in the CBCT images by matching the remaining cusps in the model; **f** The positions located via surface scanning was compared with the position in CBCT in the software 3-matic

A scan body (Regular Neck 4.8/10 mm, Straumann AG) was connected to the implant placed in the model (Fig. 1a). The models were scanned with an intraoral scanner in accordance with the manufacturer’s instruction (DL-100, Launca, Shenzhen, China) (Fig. 1b). The digital models of scan body and implant were connected virtually and simplified to a cylinder model for the convenience of following measurement (Fig. 1c, named as location component in the article) in the software (Mimics v16.0). The location component was fused into the surface scanning images using the geometry of scan body as reference (Fig. 1d). The implant part of location component was considered as the position of implant.

Each model was also scanned with an extraoral scanner (D810, 3Shape, Copenhagen, Denmark). The position of implant was measured using the methods described above. All CBCT and surface scanning procedures were performed by the same operator.

### Accuracy analysis

**Fig. 2:**
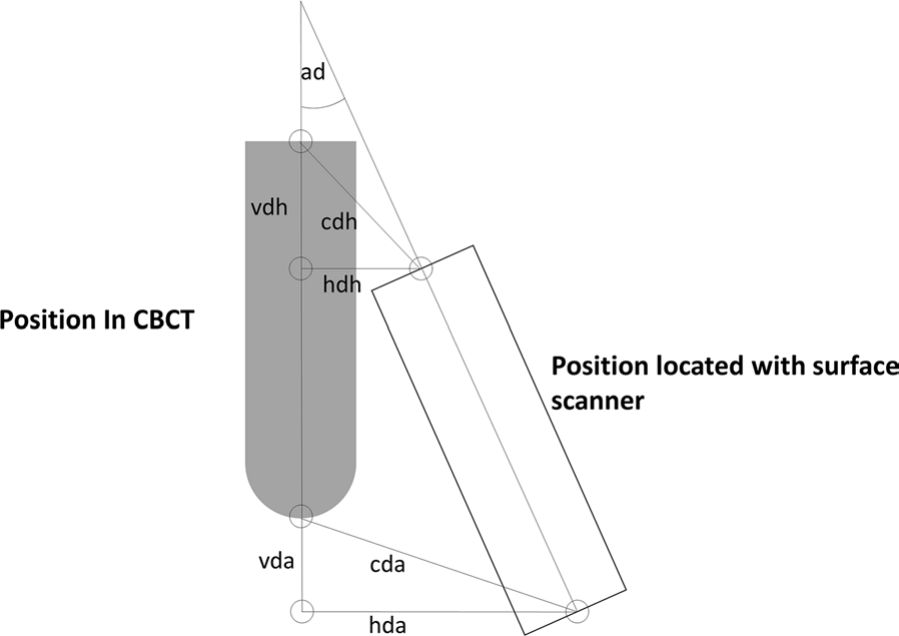
Assessment of position deviation. The parameters were defined as: cda, central deviation at apex; cdh, central deviation at hex, hda, horizontal deviation at apex, hdh, horizontal deviation at hex, vda, vertical deviation at apex, vdh, vertical deviation at hex; ad, angular deviation

The surface scanning/location component-fusing images was fused with the postoperative CBCT image using the adjacent cusps as reference (Fig. 1e). The fused images of location component, surface scanning and postoperative CBCT was uploaded into the software (3-Matic, Materialise, Leuven, Belgium) (Fig. 1f). The position deviations between implant located with surface scanning and postoperative CBCT were measured using seven parameters: central deviation at apex; central deviation at hex, horizontal deviation at apex, horizontal deviation at hex, vertical deviation at apex, vertical deviation at hex and angular deviation (Fig. 2). The values were standardized by the actual implant dimensions.

### Statistical analysis

The data was analyzed using GraphPad Prism 5 (GraphPad Software, La Jolla, CA, USA). Deviation parameters were presented as mean, maximal/minimal value (max./min.), standardized deviation (SD) and 95% confidence interval (95% CI). Statistical differences between implant deviations measured with intraoral scanner and extraoral scanner were assessed by paired t test. The level of significance was set at 0.05 in all tests.

## Results

**Fig. 3:**
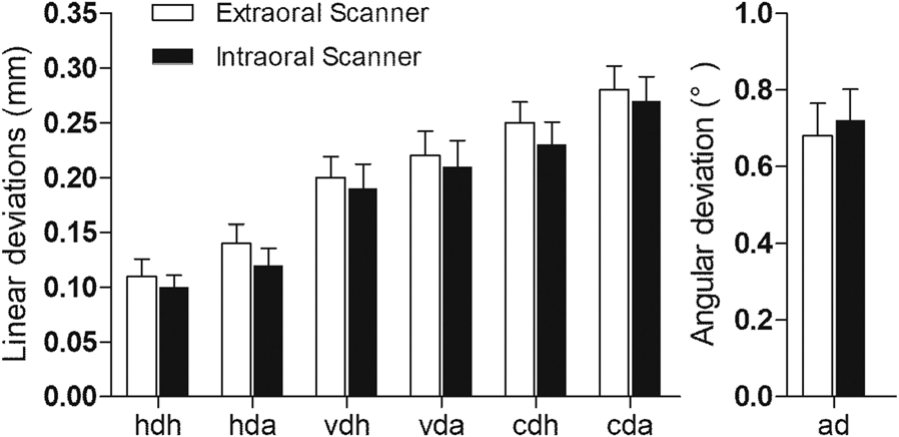
Comparison of position deviation between ES (extraoral scanner) and IS (intraoral scanner) groups. There is no significant difference between the implant deviations measured via intraoral scanner and extraoral scanner (*p* > 0.05)

In IS group, the value of cda, cdh, hda, hdh, vda, vdh and ad was 0.27 ± 0.14 mm, 0.23 ± 0.13 mm, 0.12 ± 0.10 mm, 0.10 ± 0.07 mm, 0.21 ± 0.15 mm, 0.19 ± 0.14 mm and 0.72 ± 0.52 degrees, respectively. In ES group, the value of cda, cdh, hda, hdh, vda, vdh and ad was 0.28 ± 0.14 mm, 0.25 ± 0.12 mm 0.14 ± 0.11 mm, 0.11 ± 0.10 mm, 0.22 ± 0.14 mm, 0.20 ± 0.12 mm and 0.68 ± 0.54 degrees, respectively. The implant deviations measured with intraoral scanner and extraoral scanner were summarized (Tables 1 and 2). There is no significant difference between the implant deviations measured with intraoral scanner and extraoral scanner (Fig. 3).

**Table 1:** Position deviations located with intraoral scanner

	Max	Min	Mean	S.D	95%CI	
					Lower	Upper
hdh (mm)	0.29	0.00	0.10	0.07	0.08	0.12
hda (mm)	0.38	0.00	0.12	0.10	0.10	0.16
vdh (mm)	0.58	0.01	0.19	0.14	0.15	0.24
vda (mm)	0.56	0.01	0.21	0.15	0.16	0.25
cdh (mm)	0.61	0.04	0.23	0.13	0.20	0.27
cda (mm)	0.60	0.01	0.27	0.14	0.22	0.31
ad (°)	2.24	0.04	0.72	0.52	0.57	0.88

**Table 2:** Position deviations located with extraoral scanner

	Max	Min	Mean	S.D	95%CI	
					Lower	Upper
hdh (mm)	0.41	0.00	0.11	0.10	0.08	0.14
hda (mm)	0.46	0.00	0.14	0.11	0.11	0.18
vdh (mm)	0.51	0.01	0.20	0.12	0.16	0.24
vda (mm)	0.57	0.03	0.22	0.14	0.18	0.26
cdh (mm)	0.53	0.05	0.25	0.12	0.21	0.29
cda (mm)	0.73	0.07	0.28	0.14	0.24	0.32
ad (°)	2.85	0.04	0.68	0.54	0.53	0.86

## Discussion

In this pilot study, the surface scanning was fused with the postoperative CBCT based on manually-selected fiducial points. The virtually-defined implant by scan body was compared with the implant shown in CBCT. The deviation from CBCT measurement was less than 0.3 mm linearly and 0.8° angularly. The accuracy was superior to other reported location methods alternative to postoperative radiology [5, 6].

In the presented method, the implant position was calculated based on the position of scan body. Different to the direct scanning with CBCT, the relation of implant to neighboring structures such as bony envelop, mandibular nerve and Schneiderian membrane could not be viewed directly. The indirect location is of no clinical significance unless the accuracy of the calculated position within the safe zone that is mandatory in implant surgery. A safety distance of 0.5–1 mm between the implant and vital anatomy is recommended in most literatures [12, 13]. The deviations reported in the present study were within these recommendations and should therefore be regarded as clinically acceptable if the recommendations are followed in preoperative planning. The location accuracy of our study suggested the potential application of surface scanner in assessing implant location postoperatively.

Theoretically, the direct scanning using the intraoral scanning device could result in the same and even higher accuracy than the indirect digitalization of the gypsum cast using an extraoral scanner [14]. Indirect digitalization actually collects the errors of conventional impression taking, cast pouring, impression material distortion, and optical scanning as in direct digitalization. In this study, both intraoral and extraoral scanner were used to locate the placed implant in the same in vitro model, both of which resulted in the same accuracy. Our results provided experimental evidence to clear up the doubt that intraoral and extraoral scanners may differ when applied in implant location, and suggested that the digitalization of conventional impression is no longer necessary since intraoral scanner offers accuracy of same level. More importantly, application of handheld device as intraoral scanner opens the possibility to assess implant bed preparation by scanning the indicator inserted, and to perform the implant verification immediately after insertion. The immediacy facilitates necessary intraoperative adjustment if significant deviation is detected.

Although the location accuracy shown in this model study is encouraging, it’s important to notice that the accuracy of surface scanning was generally reported to be better in vitro than that in vivo, due to better access, better space for the scanning unit, better illumination, no patient movement, and absent of saliva and blood that may lead to possible fogging of the optical unit [15]. So, whether the accuracy reported here could be realized in clinical scenarios remains to be investigated. Furthermore, the accuracy in vivo could be influenced by the length of clinical career and the region being scanned [16]. Impressions of angulated implants might also diminish the accuracy of the impression [17]. As a result, further in vivo investigations are compulsory to verify the feasibility of this method, even if acceptable accuracy was achieved in the present study.

When the proposed method used in clinical practice, the main purpose should be to assess the relation of implant and anatomic structure. The surface scanning for location is fused with preoperative planning model depicted with CBCT and surface scanning. Image fusing requires manual processing for selecting the fiducial points, which introduce errors inevitably [18]. It would be quite challenging to reduce image fusing error in clinical scenarios where the condition usually varies greatly in different cases. Beside error, manual operation is also suboptimal in daily clinical operation. Because additional trained person and computer workstation are needed during the surgery. Unfortunately, the accuracy of full-automatic fusing method is still unsatisfactory limited to the current technology [19]. More technical adaptions are in need before the practicality of implant location via surface scanner.

## Conclusion

Dental implant can be located via surface scanner. The accuracy of implant location with intraoral scanner could be acceptable for postoperative verification, but the efficiency and efficacy in clinical practice need further investigation in future.

## Data Availability

All essential data is presented in the manuscript. The step-by-step datasets and images during the current research are available from the corresponding author on reasonable request.
